# Potent and conditional redirected T cell killing of tumor cells using Half DVD-Ig

**DOI:** 10.1007/s13238-017-0429-z

**Published:** 2017-06-05

**Authors:** Philip D. Bardwell, Matthew M. Staron, Junjian Liu, Qingfeng Tao, Susanne Scesney, Gail Bukofzer, Luis E. Rodriguez, Chee-Ho Choi, Jennifer Wang, Qing Chang, Feng Dong, Cherrie Donawho, Jieyi Wang, Christine M. Grinnell, Edit Tarcsa, Charles Hutchins, Tariq Ghayur, Jijie Gu

**Affiliations:** 1 Foundational Immunology, AbbVie Bioresearch Center, Worcester, MA 01605 USA; 2 Global Preclinical Safety, AbbVie Bioresearch Center, Worcester, MA 01605 USA; 3 Oncology Discovery, AbbVie Inc., North Chicago, IL 60064 USA; 4 DMPK-BA, AbbVie Bioresearch Center, Worcester, MA 01605 USA; 5 Research and Development, AbbVie Inc., North Chicago, IL 60064 USA; 6 Present Address: Torque Therapeutics Inc., Cambridge, MA 02142 USA; 7 Present Address: Innovent Biologics Inc., Suzhou, China; 8 Present Address: Lyvgen Biopharma, Shanghai, 201203 China

**Keywords:** DVD-Ig, Half DVD-Ig, Halfbody, epidermal growth factor receptor, redirected T-cell cytotoxicity, rCTL

## Abstract

Novel biologics that redirect cytotoxic T lymphocytes (CTLs) to kill tumor cells bearing a tumor associated antigen hold great promise in the clinic. However, the ability to safely and potently target CD3 on CTL toward tumor associated antigens (TAA) expressed on tumor cells remains a challenge of both technology and biology. Herein we describe the use of a Half DVD-Ig format that can redirect CTL to kill tumor cells. Notably, Half DVD-Ig molecules that are monovalent for each specificity demonstrated reduced non-specific CTL activation and conditional CTL activation upon binding to TAA compared to intact tetravalent DVD-Ig molecules that are bivalent for each specificity, while maintaining good drug like properties and appropriate PK properties.

**Electronic supplementary material:**

The online version of this article (doi:10.1007/s13238-017-0429-z) contains supplementary material, which is available to authorized users.

## Introduction

One of the most promising clinical uses of bispecific antibody technologies is the ability of a molecule with two antigen binding specificities to redirect immune effector cells to kill tumor cells. It was first described over 25 years ago that a bispecific antibody simultaneously targeting T cells and a tumor cell surface antigen can kill target tumor cells (Staerz and Bevan, 1989). The most successful clinical programs have been the targeting of different tumor antigens (e.g., CD19 or EpCAM) by anti-CD3 redirected T cells (Baeuerle and Reinhardt, 2009).

However, an agonistic antibody can carry the danger of unwanted side-effects, with a particularly stark example of the anti-CD28 agonistic mAb TGN-1412 (Horvath et al., 2012). This antibody was designed to stimulate T cell immunity for the treatment of cancer, but severe adverse events due to activation of T cells and induction of a cytokine storm were seen in healthy volunteers. Similar safety concerns may delay or hinder development of bispecific antibodies where an anti-CD3 binding domain is paired with antibody domains against a tumor-associated antigen for redirected tumor killing by cytotoxic T cells. The TGN-1412 incident highlighted the potential risk of developing agonist antibodies to the T-cell receptor or members of the co-stimulatory receptor family. Therefore, identifying therapeutic mAbs that safely target these cell surface receptors with desired features remains a challenging task.

Blinatumomab (Blincyto^®^), an FDA-approved bispecific molecule composed of an anti-CD3 single chain variable fragment (scFv) and an anti-CD19 scFv connected by a polypeptide linker, also known as bispecific T cell engager (BiTE), showed efficacy in clinical trials for B-cell non-Hodgkin’s lymphoma and B-precursor acute lymphocytic leukemia (ALL) with manageable side effects (Nagorsen and Baeuerle, 2011). Blinatumomab is monovalent for both CD3 and CD19, which may help to avoid unwanted CD3 signaling induced by receptor dimerization or clustering, or allow for conditional CD3 clustering upon binding to the target antigen, CD19. Blinatumomab represents the first-in-class BiTE antibody approved for clinical use and provides a novel therapeutic option for patients with refractory or relapsed B cell ALL (Benjamin and Stein, 2016). One concern often being raised for BiTE molecules is the requirement for continuous intravenous (IV) infusion dosing because of the small molecular weight and rapid clearance of the molecule from circulation. Recently, a tetravalent bispecific tandem diabody (TandAb) format was constructed to address these issues. The lead molecule of TandAb format, AFM11, has two binding sites for CD3 and two for CD19, and is currently in Phase I clinical development (Reusch et al., 2015). AFM11 elicited more potent *in vitro* B cell lysis than a BiTE molecule targeting the same antigens (Molhoj et al., 2007) . In addition, TandAb with the molecular weight at approximately 100 kDa, was reported to have a half-life ranging from 18 to 23 h after IV administration in mice (Reusch et al., 2015). AFM11 therefore can be administered weekly or twice weekly in humans. However, the safety and efficacy profile of AFM11, which is bivalent for CD3 binding, is still to be determined in clinical trials.

Recently we explored the construction of a series of ‘Halfbodies’, where full-length IgG molecules are split into two equal half molecules, by structural modeling assisted mutagenesis at the antibody CH3-CH3 interface. The amino acid residues that are important for antibody C_H_3 dimerization were first described by Carter and colleagues (Dall’Acqua et al., 1998). This structure-activity relationship study of antibody C_H_3 dimers revealed that certain residues, such as T366, L368, P395, F405, Y407 and K409, played an important role in maintaining the stability of the CH3 dimers. Two separate groups have previously reported that an IgG could be converted to a monomeric IgG by introducing mutations at residues 351, 366, 368, 395, 405, 407, 409 (Ying et al., 2012), or by introducing two Asn-linked glycans at positions 364 and 407 (Ishino et al., 2013). Although previously reported formats of mAb-based monovalent-binding molecules did improve the efficacy in targeting specific cell surface targets, further development of these formats have been hindered likely due to poor manufacturing and physiochemical properties of the antibodies (Cheadle, 2006; Filpula, 2007).

We discovered that a single point mutation in the C_H_3 domain and two mutations at cysteine residues within the IgG hinge region could result in Halfbodies as well, similar to the ones generated with multiple mutations at the C_H_3 domain (Table S1 and Fig. S1). With the Halfbody format, we demonstrated the conversion of agonistic or partial antagonist molecules to pure antagonists against the cell surface target (Table S2). In addition, we extended the Halfbody technology to DVD-Ig format to generate Half DVD-Ig molecules for monovalent CD3 binding. The monovalent CD3 containing Half DVD-Ig maintained the ability to bind CD3 but was conditional with regard to their ability to initiate immune synapse formation and mediate T cell activation upon binding to tumor-associated antigen which greatly reduced non-specific cytokine release for CD3-mediated T cell redirected cytotoxicity *in vitro*. Additionally, the half DVD-Ig molecule has good expression in mammalian cells, can be purified to homogeneity, and has improved half-life compared to other monovalent antibody variants (e.g., scFv, BiTE, and Fab). Therefore, we believe that Half DVD-Ig molecular format may provide an additional option for exploring cancer treatment using redirected T cell cytotoxicity with more practical dosing regimens.

## Results

### Bispecific DVD-Ig molecules support T cell redirected killing

**Figure 1:**
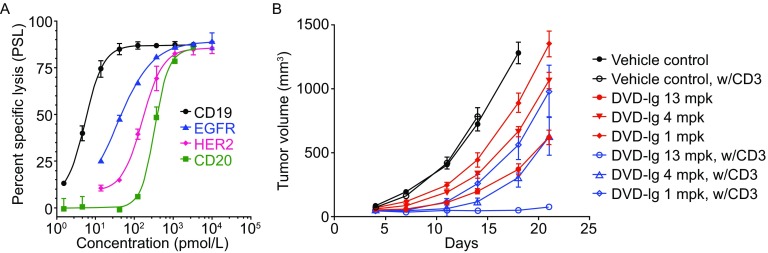
**DVD-Ig molecules elicit redirected T cell killing** ***in vitro*** **and** ***in vivo***. (A) *In vitro* CD19(●), CD20(■), EGFR(▲), HER2(♦) rCTL activity on RAJI (CD19, CD20), A431 (EGFR), and N87 (HER2) target cells were measured by cellular impedance assay. (B) *In vivo* tumor growth kinetics using A431 xenograft mouse model with dose titration of a CD3-EGFR DVD-Ig molecule (1 mpk, 4 mpk, or 13 mpk) with or without the addition of CD3-positive human T-cells

To determine if our previously described bispecific antibody DVD-Ig format (Wu et al., 2007) would efficiently redirect T cells to kill tumor cells *in vitro* we constructed anti-tumor associated antigen/anti-CD3 DVD-Ig bispecifics to a panel of well-known antigens including CD19, CD20, EGFR, and HER2 (Fig. 1A). The approximate IC_50_ values were 5, 335, 36 and 160 picomolar (pmol/L), respectively. T cell killing assays were performed *in vitro*, either with a FACS or impedance based read-out as described in the MATERIALS AND METHODS. To demonstrate the potential therapeutic benefit of the DVD-Ig format, we chose a bispecific antibody targeting EGFR to test efficacy in a mouse tumor xenograft model. As expected, anti-EGFR has single agent activity against A431 in SCID mice (Goldstein et al., 1995), but the addition of human T cells greatly suppressed A431 tumor growth (Fig. 1B). At a single dose of 13.4 milligrams per kilogram (mpk) of the 200 kDa DVD-Ig (equivalent to 10 mpk of a 150 kDa antibody), growth of A431 tumor cells was completely inhibited. The potency of the EGFR-CD3 DVD-Ig was comparable to what was previously reported for BiTE scFv bispecific antibodies (Lutterbuese et al., 2010).

### Construction, expression, and biochemical characterization of Half DVD-Ig molecules

Previously we demonstrated the generation of stable Half Ig molecules by introducing P395A, F405R, Y407R, and K409D mutations, at the C_H_3 domain to disrupt C_H_3 homodimerization and C226S and C229S mutations, at hinge region, to eliminate the two inter-chain disulfide bonds of the immunoglobulin molecules (Table S1). We further demonstrated that a single point mutation in the C_H_3 domain and two mutations at cysteine residues within the IgG hinge region could result in Halfbodies as stable as the ones with multiple mutations at C_H_3 domain (Table S1 and Fig. S1). With the Halfbody format, we achieved conversion of a cMet agonistic (or partial antagonist) antibody, to a pure antagonist. This was demonstrated in a cMet phosphorylation assay, where unlike the parental antibody, the Halfbodies neutralized HGF-induced cMet phosphorylation. Furthermore the halfbodies, but not the parental anti-cMet antibody, inhibited tumor cell proliferation (Table S2).

**Figure 2:**
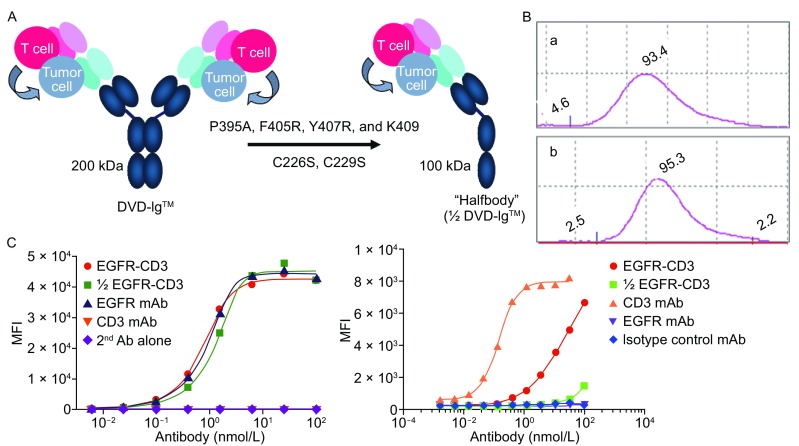
**Half DVD-Ig design and characterization**. (A) Mutagenesis at C_H_3 and hinge region converted anti-EGFR/anti-CD3 DVD-Ig into a Half DVD-Ig molecule. (B) (a) Intact anti-EGFR/anti-CD3, shown for comparison, and (b) Half DVD-Ig protein can be produced and purified to homogeneity with approximate 95% monomer percentage analyzed by size exclusion chromatography. (C) Binding of Half DVD-Ig to EGFR on A431 cells (left) and CD3 on Jurkat cells (right)

To understand if we are able to generate Half DVD-Ig molecules to study the effect of anti-CD3 binding valency on redirected T cell cytotoxicity for tumor killing *in vitro*, we generated a Half anti-EGFR (outer domain)/anti-CD3 (inner domain) DVD-Ig molecule connected using a long-long (L-L) linker by including four mutations at C_H_3 domain and two cysteine mutations at the hinge region (Fig. 2A). The Half DVD-Ig molecule was expressed in HEK293 cells and purified to homogeneity using protein A affinity chromatography. The half DVD-Ig molecule showed molecular size at approximately 100 kDa and 95% monomer analyzed by size exclusion chromatography (Fig. 2B). The full-length DVD-Ig that is bivalent for both EGFR and CD3, where anti-EGFR is on the outer domain and anti-CD3 is on the inner domain with L-L linker, demonstrated comparable binding to EGFR as the anti-EGFR mAb, but showed reduced binding to CD3 compared to an anti-CD3 mAb. This is likely due to steric hindrance of the outer domain since when CD3 is moved to the outer domain (and EGFR is moved to the inner domain), CD3 binding is recovered to anti-CD3 mAb levels (data not shown). The Half DVD-Ig also showed binding to the EGFR expressing tumor cell line A431 but the binding to CD3 expressed on Jurkat cells was greatly reduced compared to DVD-Ig (Fig. 2C), which is most likely due to a significant loss in binding associated with a monovalent anti-CD3.

### Anti-EGFR/Anti-CD3 Half DVD-Ig molecules for T cell redirected cytotoxicity

One potential issue with utilizing full length antibody constructs with Fc domains is the interaction of anti-(tumor associated antigen) molecules/anti-CD3 with Fc-receptors and the potential for cytokine release by non-specific T cell activation. In addition, bivalent CD3 binding by a full-length antibody might cross-link CD3 and have T cell mitogenic activity without the engagement of tumor antigens. Together, these T cell activation mechanisms can result in cytokine release syndrome (CRS), sometimes with potentially serious adverse events (Sathish et al., 2013).

**Figure 3:**
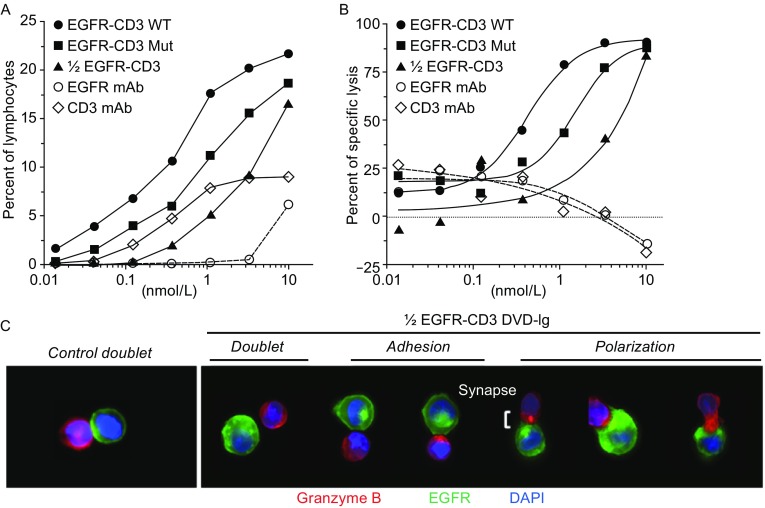
**DVD Halfbodies promote conditional T cell activation and redirected T cell killing of tumor cells** ***in vitro***. (A) FACS analysis of CD8+CD25+ of huPBMC taken from rCTL assay. (B) Potency of anti-EGFR/anti-CD3 DVD-Ig molecules with huIgG1-wild type (EGFR-CD3 WT, circles), huIgG1-mutant (EGFR-CD3 Mut, squares), and anti-EGFR/anti-CD3 Half DVD-Ig molecule (1/2 EGFR-CD3, triangles) in a redirected cytotoxicity (rCTL) assay. Control anti-EGFR mAb (EGFR mAb, open circles) and anti-CD3 (CD3 mAb, open diamonds) mAbs were included in assays. Data are representative of 3 separate experiments from 1 huPBMC donor. (C) Anti-EGFR/Anti-CD3 Half DVD-Ig molecule promote immune synapse formation between T cells (granzyme B+, red) and A431 tumor cells (green), and induced T cell polarization of granzyme B (red) as visualized by Amnis Imagestream

Therefore, we reasoned that a Half DVD-Ig molecule that is only capable of monovalent interaction with the CD3 receptor on T cells and potentially minimal (or no) Fc function may have the potential to mitigate non-specific T cell activation. To test this hypothesis, we took the well-known OKT3 anti-CD3 variable domains and combined them with domains derived from Cetuximab (anti-EGFR) to create three different formats of DVD-Ig molecules, i.e., huIgG1-wild-type, huIgG1-mutant (L234A, L235A), and the anti-EGFR/anti-CD3 Half DVD-Ig molecule described above. We then compared these molecules in redirected cytotoxicity (rCTL), T-cell activation and cytokine release assays. All three formats support rCTL activation as demonstrated by an upregulation of the high affinity IL-2R alpha chain CD25 on CD8^+^ T cells (Fig. 3A), and killing of EGFR-positive A431 cells *in vitro* (Fig. 3B). As expected, the potencies were reduced with the huIgG1-mutant likely due to reduced FcγR interactions with peripheral blood mononuclear cells (PBMCs), and with the Half DVD-Ig format likely due to monovalent binding for T cell activation. Consistent with killing of EGFR-positive A431 cells *in vitro*, the addition of Half DVD-Ig was specifically able to promote capping of the T cell receptor (adhesion) and re-direction of granzyme B (polarization) toward EGFR-positive A431 cells, indicative of a proper immune synapse (Fig. 3C).

**Figure 4:**
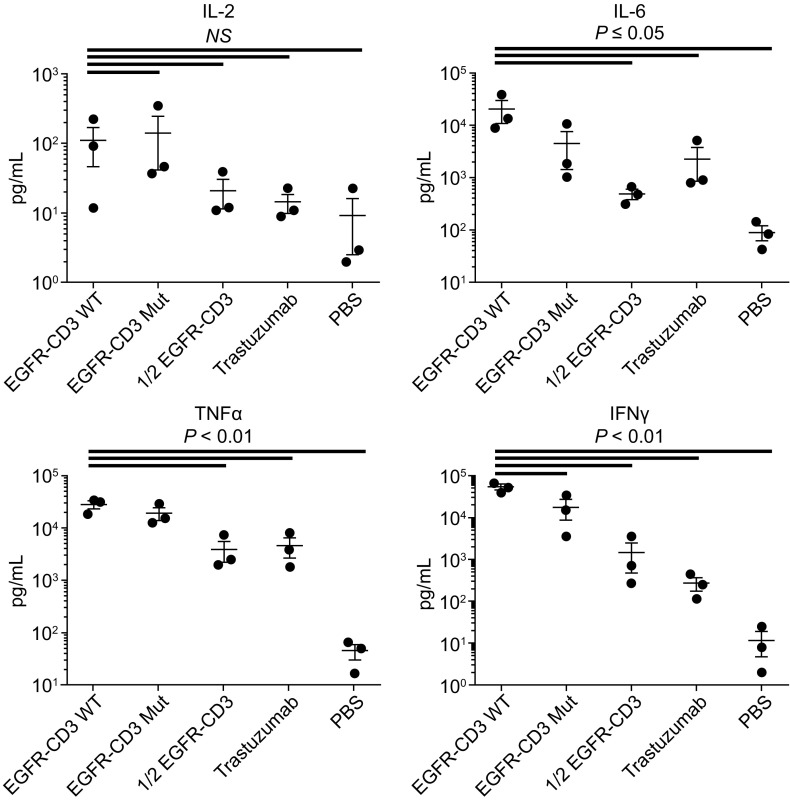
**The role of DVD-Ig monovalent target binding on T cell function**. Cytokines were measured by MSD in supernatants taken from huPBMC cultures incubated with the indicated antibody or DVD-Ig molecules (coated on plate at 1 μg/well) for 48 h. Different donors (*n* = 3) were simultaneously assayed. Statistic *P*-value was calculated using one-way ANOVA. *NS,* not significant

Next, we compared the ability of DVD-Ig and Half DVD-Ig to promote activation and cytokine secretion in primary human PBMCs. Antibody or DVD-Ig proteins were coated in the well before culturing human PBMCs to compare cytokine release induced by DVD-Ig with wild type or mutant Fc and Half DVD-Ig molecule (Fig. 4). Three separate donors were compared for cytokine release after forty-eight hours of culture. Trastuzumab served as the negative antibody control and PBS was the media control. Of note, trastuzumab treatment of human PBMCs, similar to any human IgG, does elicit background levels of cytokine release, which is not known to induce significant CRS in the clinic (Finco et al., 2014). The hallmarks of CRS, after treating a patient with an agent like an anti-CD3 antibody, are the transient but significant increases in the cytokines TNFα, INFγ, IL-2 and IL-6 (Sathish et al., 2013). Strikingly, the Half DVD-Ig format is more similar to trastuzumab in inducing lower cytokine release than either the wild type Fc or mutant Fc DVD-Ig formats. These results may be due to both a reduction in FcγR binding of the Halfbody compared to IgG1 mutant and monovalent binding to CD3.

**Figure 5:**
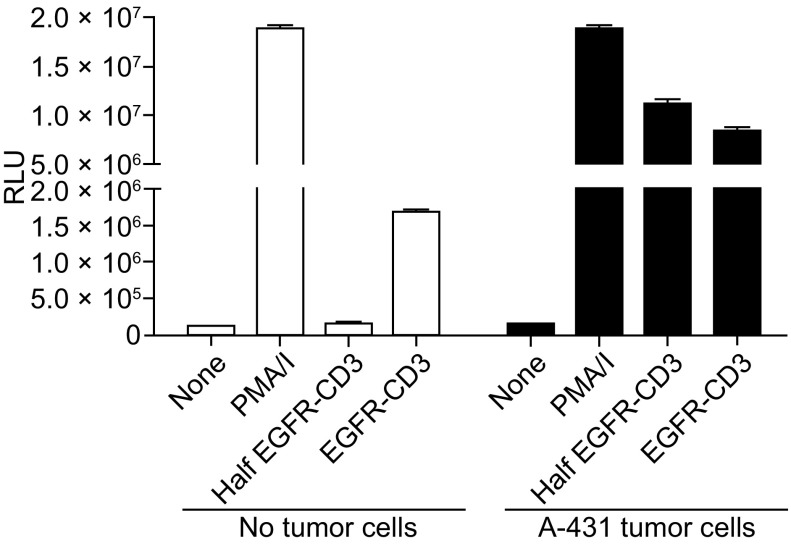
**Conditional activation of T cells by anti-EGFR/anti-CD3 Half DVD-Ig**. Activation of NFAT reporter in Jurkat T cells by anti-EGFR/anti-CD3 DVD-Ig and anti-EGFR/anti-CD3 Half DVD-Ig molecules in the presence or absence of A-431 tumor cells that express EGFR. PMA plus ionomycin (PMA/I) stimulation is included as a control

To further examine the role of CD3 binding valency in a non-specific or ‘conditional’ T cell activation, we examined the ability of DVD-Ig or Half DVD-Ig to stimulate a NFAT reporter in Jurkat T cells in the presence or absence of tumor cell associated antigen. The DVD-Ig molecule led to activation of a sensitive NFAT reporter gene in Jurkat T cells independent of tumor cells, that is, the DVD-Ig could initiate TCR signaling even in the absence of tumor associated antigen. However, the Half DVD-Ig molecule was only able to efficiently trigger TCR signaling in the presence of tumor cell antigen which likely functioned as an anchor to help stabilize CD3 binding to the T cells and promote conditional TCR crosslinking (Fig. 5). This data suggests that a Half DVD-Ig molecule containing monovalent anti-CD3 and minimal Fc receptor-mediated effector functions may be advantageous for indications where a reduced risk for CRS would be beneficial.

### Pharmacokinetic properties of Halfbodies

The PK properties of Halfbodies were evaluated in Sprague Dawley rats. Following 4–5 mg/kg intravenous (IV) dosing, intact anti-EGFR/anti-CD3 DVD-Ig protein showed a half-life of 7–8 days. However, anti-EGFR/anti-CD3 Half DVD-Ig protein had a half-life of approximately 31 h (Table 1). The interaction between the Fc region of an IgG molecule and the neonatal Fc receptor (FcRn) plays a critical role in determining antibody half-life (Roopenian and Akilesh, 2007). Three histidine residues located at the C_H_2 and C_H_3 junction region of IgGs regulate IgG binding to FcRn at acidic pH (<6.5) and release from FcRn at neutral pH (~7.4). This pH-sensitive FcRn binding protects IgGs from catabolism and contributes to their long serum half-life (Roopenian and Akilesh, 2007). Although spatially the mutations introduced to disassociate C_H_3 dimerization are distant from the FcRn binding region in C_H_2-C_H_3, Half DVD-Ig molecules that have the F405R or other C_H_3-mutations exhibited significantly reduced serum half-life. The shorter half-life may be the consequence of reduced pH-sensitive binding to FcRn due to the mutations, the monomeric Fc region resulting in less binding, or there may be other explanations and further studies may lead to a better understanding. Nevertheless, the Half DVD-Ig molecule has an improved PK profile compared to other monovalent antibody variants such as scFv, BiTE, and Fab (Weisser and Hall, 2009).

**Table 1:** Pharmacokinetic profile of Half DVD-Ig compared to DVD-Ig molecules in Sprague Dawley rats

Ig	T_1/2_^#^(h)	Vss (mL/kg)	CL (mL/h/kg)	AUC_0-t_ (h*mg/mL)
Anti-EGFR/anti-CD3 DVD-Ig protein	160.0	254.0	1.8	2.7
Anti-EGFR/anti-CD3 Half-DVD-Ig protein	31.0	69.2	4.9	1.0

5 mg/kg IV dosing for DVD-Ig protein and Half-DVD-Ig protein. 5 animals per group

^#^Harmonic mean and pseudo-standard deviation used

## Discussion

One of the most promising clinical applications of bispecific technologies is the redirection of immune effector cells to kill tumor cells. A good review of the clinical history of immune cell rCTL mediated by various bispecific formats has been published by Lum and Thakur (2011). The first successful clinical program using redirected T cells was accomplished by Fresenius Biotech and Trion Pharma’s Catumaxomab (Removab^®^), which was approved in the EU in 2009 for malignant ascites. Catumaxomab was made of a rat/mouse quadroma which required devising a purification scheme to isolate pure heteroconjugates with specificity to CD3 on one arm and EpCAM on the other. Notably, catumaxomab was reported to have intact Fc effector function (Chelius et al., 2010). Since this molecule contains rat and mouse proteins, developing anti-drug antibody response in the clinic limited broader applicability of the approach. A major breakthrough in the field occurred when Micromet was able to demonstrate the efficacy and safety of blinatumomab as a B cell lymphoma therapy (Baeuerle and Reinhardt, 2009). Blinatumomab is a scFv-based bispecific T-cell engager (BiTE) molecule containing anti-CD3 and anti-CD19 domains. BiTE molecules have short half-lives, so patients must undergo continuous infusion by micropumps. BiTE molecules have monovalent binding domains and do not engage Fc receptors since they do not have an Fc domain. Therefore, BiTE molecules are thought to minimize the risk of CRS (Loffler et al., 2000).

The Halfbody format also has the potential to minimize the risk of a cytokine storm by not triggering non-specific T cell activation. It is well understood that crosslinking of the T cell receptor with anti-CD3 antibodies causes rapid T cell activation and can lead to serious clinical adverse events (Sgro, 1995). Bluestone and colleagues attempted to mitigate CRS for the treatment of type-I diabetes by making a chimeric antibody with an OKT3 variable domain and a huIgG1-mutant (L234A, L235A) constant region to prevent FcγR binding (Chatenoud and Bluestone, 2007). Although the *in vitro* lack of cytokine release looked promising, this result did not translate clinically because CRS was observed in patients, even though it was less severe than the parental wildtype OKT3 (Kaufman and Herold, 2009). We have shown that a half anti-EGFR/anti-CD3 DVD-Ig protein can support sub-nmol/L rCTL activation while minimizing the risk of cytokine release in our *in vitro* assay. It is still unclear whether high levels of cytokine production may be necessary for efficacy of an rCTL agent and what the clinically tolerable level of cytokine release might be (Lum and Thakur, 2011). Further studies are needed to understand if our results would translate clinically. The balance of safety and potency must be managed, and the Halfbody format provides a promising option that could be used for appropriate cancer indications.

Although the mean serum half-life of Halfbodies, estimated to be approximately 31 h, is much shorter than that of full-length antibodies, it is still a significant improvement over half-life of BiTE, Fab or scFv molecules (Chapman et al., 1999; Cheadle, 2006). This could be due to the fact that the molecular size of Halfbodies is larger than the threshold of renal clearance of proteins (McAleese and Eser, 2012). TandAb, a tetra-valent bispecific scFv antibody format with molecular size of ~110 kDa, is reported to have a serum half-life similar to Halfbodies reported here. With the estimated 12–24 h half-life in cynomolgus monkey, AFM13, an anti-CD16A X anti-CD30 TandAb, was enabled for a Phase I trial with weekly infusion dosing and the clinical PK results were reported to be promising (Rothe et al., 2015). If our observed rat PK correlates with human PK, we hypothesize that our Halfbody format would show a similar improvement in PK properties over scFv molecules. With advantages of monovalent-targeting, reduced Fc function, ease of expression and purification, preferred drug-like properties, and superior profile in serum half-life over other monovalent antibody fragments, Halfbody molecules may offer a promising novel therapeutic option.

## Materials and Methods

### Generation and characterization of Half DVD-Ig molecules

The cDNA sequences of anti-cMet variable domains were cloned from mouse hybridoma HB-11895 (5D5.11.6) (ATCC, Manassas, VA) with a mouse Ig primer set (Novagen, Cat # 69831-3). For cMet Halfbody molecules, mutations such as C226S, C229S, T366F, L368F, P395A, F405R, Y407R, K409D were introduced into the heavy chain hinge region using standard mutagenesis techniques. The variable domain sequences of the anti-CD3 antibody were derived from the OKT3 antibody and those of the anti-EGFR antibody were derived from cetuximab. Inner and outer domains were connected using a long-long linker (L-L): VH1-VH2 Linker, ASTKGPSVFPLAP (13a.a); VL1-VL2 Linker, TVAAPSVFIFPP (12a.a.). AbbVie-proprietary pHybE vectors with corresponding huIgG1 heavy chain and kappa light chain were used to transiently transfect 293-6E cells in Freestyle 293 medium (Invitrogen, Carlsbad, CA). Media containing the secreted protein was harvested 6–7 days post transfection and purified using protein A chromatography (Invitrogen, Carlsbad, CA) according to the manufacturer’s instructions. The proteins were then dialyzed into PBS buffer, pH 7.2 (Invitrogen, Carlsbad, CA). The monomer percentage of Half DVD-Ig proteins was analyzed by SDS-PAGE and size exclusion chromatography. Binding of anti-EGFR/anti-CD3 DVD-Ig and half DVD-Ig molecules to CD3 and EGFR was analyzed by FACS.

### *In vivo* redirected cytotoxicity tumor models

Immune compromised female SCID mice were purchased from Charles Rivers (Wilmington, MA). Animals were acclimated to the animal facilities for a period of at least one week prior to commencement of experiments. Animals were kept in the light phase of a 12 h light:12 h dark schedule (lights on 0600 h). All experiments were conducted in compliance with AbbVie’s Institutional Animal Care and Use Committee and the National Institutes of Health Guide for Care and Use of Laboratory Animals guidelines in a facility accredited by the Association for the Assessment and Accreditation of Laboratory Animal Care. A431 cells were obtained from the Ludwigshafen Institute, cells were grown between passage 3–6 *in vitro* in RPMI 1640 media with 10% FBS. Donor T-cells were purchased from Astarte Biologicals, Bothell, WA. T-cells were positive selected and counted using a flow cytometer by Astarte Biologicals. T-cells were brought up from frozen in RPMI 1640, 10% FBS, 2 mmol/L L-glutamine, 55 mmol/L 2-BME and 30 IU/mL recombinant human IL-2, and maintained in the incubator for 24 h before use. Tumor and T-cell viability was assessed by trypan blue exclusion and later mixed in a 50 mL conical tube prior to inoculation, achieving a 1:1 effector:tumor ratio (E:T). A total of 1 × 10^6^ viable A431 cells and/or T-cells were inoculated subcutaneously into the right flank of female SCID mice on day 0. The total injection volume was 0.1 mL and was composed of a 1:1 mixture of S-MEM and matrigel (Corning, MA). The A431 tumor model provided 100% tumor take and robust tumor growth, making it feasible for evaluation as an early start tumor model. In the early start tumor model, dosing of reagents started 1 h post tumor inoculation. Tumor measurements were collected twice a week and animals were removed from the study once tumors reached ≤1,500 mm^3^.

### *In vitro* redirected cytotoxicity assay

Raji, A431 or N87 (ATCC, Manassas, VA) target (T) cells were allowed to adhere to ACEA xCELLigence 96-well plates (ACEA Bio, San Diego, CA) overnight and cell indexes are periodically monitored. Cell index is a measurement of electrical impedance and reflects the viability of adherent cells. Human PBMC effector cells (E) were then plated at an E:T ratio of 10:1. The testing samples were appropriately diluted to obtain concentration-dependent titration curves. The cell indexes of targets in the DVD-Ig protein treated samples were divided by the cell indexes of control targets (no treatment) to calculate percent specific lysis. The data was graphed and EC_50_s were calculated in Prism (Graphpad Software, San Diego, CA). T cell activation was monitored by harvesting cells from 96 well plates and subjected to FACS analysis (FACSCanto II, BD Biosciences, San Jose, CA) after staining with anti-CD4, CD8, CD25, and CD69 fluorescently-labeled antibodies (eBioscience, San Diego, CA).

### Cytokine release huPBMC assay

A similar assay has been previously described (Finco et al., 2014). Briefly, test antibodies were coated onto a 96-well polypropylene plate at 1 μg/mL for 90 min at 37°C. The plate was rinsed and human PBMC were added at 10^5^ cells/well. After 48 h of culture, supernatants were harvested and assayed for cytokines using commercially available kits (Meso Scale Discovery, Rockville, MD).

### Visualization of immune synapse formation

Purified CD3-positive T cells were stimulated with anti-CD3 plus anti-CD28 for three days to generate T cell blasts that express stored granzyme-B. T cell blasts were subsequently co-cultured with A431 cells in the presence or absence of Half DVD-Ig for 4–6 h to allow for immune synapse to form. Cells were fixed, washed, permeabilized, and stained with fluorescent antibodies against EGFR and granzyme-B. DAPI was used to visualize the nucleus. Conjugates were visualized on Amnis ImageStream by gating CD3 and EGFR doublets. Representative image overlays were cropped together to illustrate the various stages of synapse formation.

### Jurkat reporter assays

Jurkat-NFAT luciferase reporter cells (Promega, Madison, WI) that express luciferase under the control of an NFAT transcription factor binding site were stimulated with 5 nmol/L Half DVD-Ig or 5 nmol/L DVD-Ig in the presence or absence of A431 tumor cells that express an abundance of the tumor-associated antigen, EGFR. After 4–6 h, the co-cultures were lysed with SteadyGlo luciferase substrate (Promega) and relative luciferase activity was read using a luminometer.

### Pharmacokinetics in rats

Pharmacokinetic (PK) properties of Halfbodies were determined in Sprague-Dawley rats purchased from Charles River Laboratories (Wilmington, MA). Male rats were dosed intravenously with a single dose of 4 mg/kg of test Halfbody molecules. Blood samples for serum concentration analysis were collected via the tail vein in serum separator tubes at various time points. The serum samples were analyzed on a MSD Sector Imager 6000 (Meso Scale Discovery, Gaithersburg, MD) in a chemiluminescent MSD method using biotinylated cMet for capture and sulfo-tagged anti-human IgG Kappa for detection. Sample concentrations were calculated by XLfit4 software version 2.2.1 Build 16 (Microsoft Corporation, Redmond, WA). PK parameters were calculated using Winonlin software version 5.0.1 (Pharsight Corporation, Mountain View, CA). All rodents were obtained from vendors and were handled under procedures approved by the AbbVie Institutional Animal Care and Use Committee Association, which is accredited by the Association for Assessment and Accreditation of Laboratory Animal Care.

## ^Electronic supplementary material^

^Below is the link to the electronic supplementary material.^

^Supplementary material 1 (PDF 201 kb)^
