# Supplementary material for: Potent and conditional redirected T cell killing of tumor cells using Half DVD-Ig
Source: Protein Cell. 2017 Jun 5;9(1):121–9. doi: 10.1007/s13238-017-0429-z (PMC5777973; doi:10.1007/s13238-017-0429-z)
Supplement: Supplementary file 1 — Supplementary material 1 (PDF 201 kb) [file 13238_2017_429_MOESM1_ESM.pdf]

Supplementary Table S1: Minimum CH3 Mutations Required for Maintaining Halfbody Status

Ig	Hinge mutation	CH3 Mutation	Halfbody Percentage#
cMet mAb	None	None	0
cMet-mut1	C226S, C229S	None	0
cMet-mut2	None	T366F, L368F, P395A, F405R, Y407R, K409D	56
cMet-mut3	C226S, C229S	T366F, L368F, P395A, F405R, Y407R, K409D	82
cMet-mut4	None	P395A, F405R, Y407R, K409D	92
cMet-mut5*	C226S, C229S	P395A, F405R, Y407R, K409D	97
cMet-mut6	C226S, C229S	P395A, F405R, Y407R	99
cMet-mut7	C226S, C229S	F405R, Y407R, K409D	98
cMet-mut8	C226S, C229S	P395A, Y407R, K409D	70
cMet-mut9	C226S, C229S	P395A, F405R, K409D	99
cMet-mut10	C226S, C229S	P395A, F405R	95
cMet-mut11	C226S, C229S	P395A, Y407R	97
cMet-mut12	C226S, C229S	P395A, K409D	95
cMet-mut13	C226S, C229S	F405R, Y407R	96
cMet-mut14	C226S, C229S	F405R, K409D	98
cMet-mut15	C226S, C229S	Y407R, K409D	51
cMet-mut16	C226S, C229S	P395A	0
cMet-mut17**	C226S, C229S	F405R	98
cMet-mut18	C226S, C229S	Y407R	94
cMet-mut19	C226S, C229S	K409D	92

* anti-cMet Halfbody-1. ** anti-cMet Halfbody-2. # Determined by SEC.

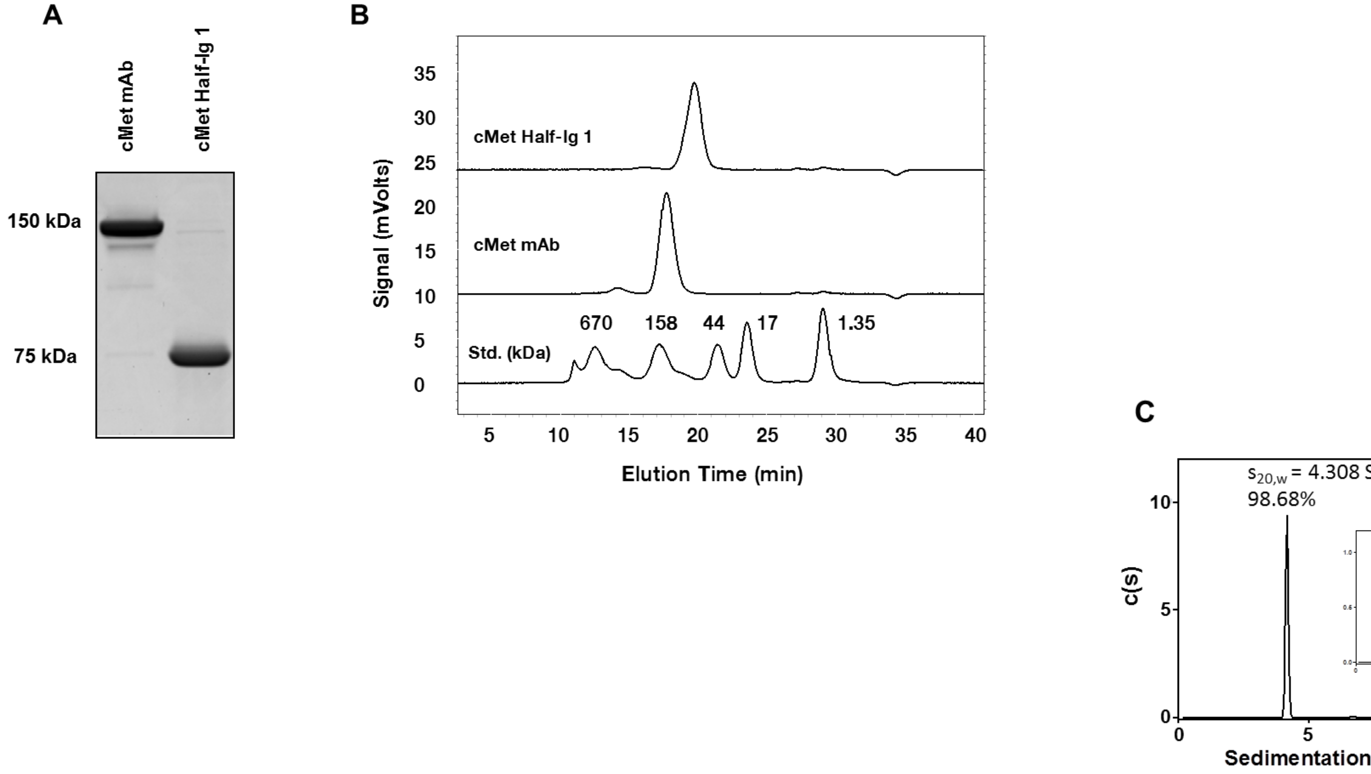

***Supplementary Figure S1. Physicochemical characterization of anti-cMet Halfbody-1. A**, Non-reducing SDS-PAGE analysis of anti-cMet Halfbody-1. **B**, Analysis of anti-cMet Halfbody-1 by size exclusion chromatography (SEC). **C**, In vitro stability analysis of anti-cMet Halfbody-1 by sequential sedimentation velocity analytical ultracentrifuge (SV-AUC) and physical mixing. Only the scanning data after the 3rd sedimentation velocity run is shown. **D**, Overlaying SEC scanning before and after three AUC runs.*

Supplementary Table S2: Functional Characterization of cMet Halfbody Molecules.

Test article	cMet Binding by ELISA	Effect on HGF-dependent cMet activation (A549 cells)	Effect on HGF-independent cMet activation (SNU5 cells)

	EC50 (nM)	Maximum OD_{450}	Inhibition of HGF-induced cMet phosphorylation (%)	Testing article-induced cMet phosphorylation (%)*	Inhibition of cancer cell proliferation (%)
cMet Halfbody-1	0.96	1.3	96	3	25
cMet Halfbody-2	1.08	1.3	100	5	24
cMetMab	0.29	2.6	82	52	(18)**
HGF				100	

Supplementary Table S2: The impact of anti-cMet Halfbody molecules on phosphorylation of cell surface cMet was determined by cMet phosphorylation assay using A-549 adenocarcinoma human alveolar basal epithelial cells (Table 3). As expected, HGF, a cMet ligand, induced strong cMet phosphorylation through the HGF/cMet signal pathway. When the parental cMet antibody was co-incubated with HGF, a moderate level of cMet phosphorylation (18%) was observed, indicating that the cMet antibody was a partial antagonist to HGF-induced cMet phosphorylation. In contrast, both anti-cMet Halfbody-1 and -2 exhibited potent antagonistic activities in the cellular assay, by completely neutralizing HGF-induced cMet phosphorylation. The parental anti-cMet antibody, due to its bivalent binding to cell surface cMet, acted as a partial agonist by inducing 52% cMet phosphorylation (normalized to maximal HGF signal) by itself in the absence of HGF. Notably, both anti-cMet Halfbodies exhibited minimum agonistic activity, if any, in induction of cMet phosphorylation in the absence of HGF. As expected, the anti-cMet Halfbodies, as well as the parental anti-cMet mAb, showed little effect on the constitutively active cMet in SNU5 gastric cancer cells with an amplified MET gene. These data suggest that Halfbodies have the potential to be applied to various monovalent targeting biotherapeutics where a reduction of agonist activity or a pure antagonist is desired.
*Data was normalized to HGF signal. ** () = stimulation.
